# Early response in cognitive-behavior therapy for syndromes of medically unexplained symptoms

**DOI:** 10.1186/s12888-017-1351-x

**Published:** 2017-05-25

**Authors:** Maria Kleinstäuber, Michael J. Lambert, Wolfgang Hiller

**Affiliations:** 1 Division of Clinical Psychology and Psychotherapy, Philipps-University, Gutenbergstr. 18, D-35037 Marburg, Germany; 2 Psychological Medicine, School of Medicine, Faculty of Medical and Health Sciences, University of Auckland, Hospital Bldg. 599, 2 Park Rd, Grafton Auckland, Auckland, 1023 New Zealand; 3 Department of Psychology, Brigham Young University, Provo, USA; 4 Department of Clinical Psychology and Psychotherapy, Johannes Gutenberg-University, Mainz, Germany

**Keywords:** Early response, Cognitive-behavior therapy, Medically unexplained symptoms, CBT, MUS

## Abstract

**Background:**

Early dramatic treatment response suggests a subset of patients who respond to treatment before most of it has been offered. These early responders tend to be over represented among those who are well at termination and at follow-up. Early response patterns in psychotherapy have been investigated only for a few of mental disorders so far. The main aim of the current study was to examine early response after five therapy-preparing sessions of a cognitive behavior therapy (CBT) for syndromes of medically unexplained symptoms (MUS).

**Methods:**

In the context of a randomized, waiting-list controlled trial 48 patients who suffered from ≥3 MUS over ≥6 months received 5 therapy-preparing sessions and 20 sessions of CBT for somatoform disorders. They completed self-report scales of somatic symptom severity (SOMS-7 T), depression (BDI-II), anxiety (BSI), illness anxiety and behavior (IAS) at pre-treatment, after 5 therapy-preparing sessions (FU-5P) and at therapy termination (FU-20 T).

**Results:**

The current analyses are based on data from the treatment arm only. Repeated measure ANOVAs revealed a significant decrease of depression (*d* = 0.34), anxiety (*d* = 0.60), illness anxiety (*d* = 0.38) and illness behavior (*d* = 0.42), but no change of somatic symptom severity (*d* = −0.03) between pre-treatment and FU-5P. Hierarchical linear multiple regression analyses showed that symptom improvements between pre-treatment and FU-5P predict a better outcome at therapy termination for depression and illness anxiety, after controlling for pre-treatment scores. Mixed-effect ANOVAs revealed significant group*time interaction effects indicating differences in the course of symptom improvement over the therapy between patients who fulfilled a reliable change (i.e., early response) during the 5 therapy-preparing sessions and patients who did not reach an early reliable change. Demographic or clinical variables at pre-treatment were not significantly correlated with differential scores between pre-treatment and FU-5P (−.23 ≤ *r ≤ .*23).

**Conclusions:**

Due to several limitations (e.g., small sample size, lack of a control group) the results of this study have to be interpreted cautiously. Our findings show that reliable changes in regard to affective-cognitive and behavioral variables can take place very early in CBT of patients with distressing MUS. These early changes seem to be predictive of the outcome at therapy termination. Future studies are needed in order to replicate our results, and to identify mechanisms of these early response patterns in somatoform patients.

**Trial registration:**

ISRCTN. ISRCTN17188363. Registered retrospectively on 29 March 2007.

## Background

The term “early response” originates from pharmacological research, in particular research of inexplicable relapse during continuation treatment [1]. The Columbia Group led by Frederic M. Quitkin [2] defined the phenomenon of the “true-drug initial response pattern” which means that a *delayed* persistent response predicts a positive long-term outcome of an antidepressant treatment. In the contrary, an *early* response going along with patterns of fluctuating improvement is rather associated with relapse [3] and rather attributable to placebo effects [2].

In psychotherapy research only a few studies [4–8] have investigated the timing of response in psychotherapy. In contrast to pharmacotherapy, they demonstrated that early response patterns are related to better intermediate and long-term outcomes. One of the earliest studies on rapid response in psychotherapy was conducted by Fennell and Teasdale [4]. The authors compared a sample of patients with major depressive disorder (MDD) who responded to cognitive-behavior therapy (CBT) with a median change in depressive symptoms (46%) or more with MDD patients whose response was lower than a median improvement before the fourth therapy session. The results showed that the rapid responders maintained their early therapy gains throughout the active treatment period. Moreover, those who demonstrated an early therapy response continued to do better until the end of the treatment than those who initially hardly improved. Patients who were below the median change of depressive symptoms after the first three sessions had outcomes at the end of therapy which were similar to those of control participants who received treatment as usual (TAU). Finally authors could demonstrate that the extent of early change in depressive symptoms strongly predicted post-treatment outcome in the CBT but not in the TAU control group.

In another study Renaud et al. [5] demonstrated (in a group of depressive adolescents) that early responders compared to individuals whose score did not change or deteriorated after the first two treatment weeks had better outcomes at the end of therapy and at a 1- and 2-year follow-up. This finding was similar across three different kinds of therapy (CBT, systemic behavioral family therapy, and nondirective supportive treatment). Similarly Haas et al. [6] showed in an outpatient psychotherapy sample of 147 patients with mixed diagnoses of mental disorders that a rapid response across sessions 1 through 3 predicted better long-term outcomes, even after analyses controlled for initial symptom severity. Furthermore there are two studies [7, 8] in which patterns of response in early therapy sessions were examined as a function of patients’ pre-treatment symptom severity.

Lutz et al. [7] examined a completer sample of *N* = 162 depressive patients treated with different treatment protocols (cognitive behavior therapy, interpersonal therapy, imipramine plus clinical management, pill-placebo plus clinical management) were analyzed with growth mixture models in order to identify meaningful patterns of early change of depression severity. Type of treatment had low explanatory power for the change of depressive symptoms at termination and follow-up. In contrast, patterns of early change as well as pre-treatment overall symptom severity were significant predictors of short- and long-term outcomes. In particular results showed that patients with moderate to severe depression and rapid early improvement had the highest rate of reliable improvement (100%) at both 16- week and 18-month follow-up. In contrast, the lowest rate of improvement at termination (34%) was found in patients who had a mild to moderate level of depression at pre-treatment and who demonstrated a moderate improvement during early therapy sessions. However, these patients showed further improvement during the follow-up phase. In another study by Lutz et al. [8] a large sample of psychotherapy outpatients with mixed diagnoses was analyzed with growth mixture models. According to their level of pre-treatment depression patients were assigned to one of three subgroups. Group 1 indicated mild, group 2 moderate, and group 3 severe depressive symptoms at pre-treatment. For each group the authors identified 4 different response patterns. Whereas some of these response patterns were less similar between the three groups, there was one pattern which appeared in all three subgroups and which was characterized by only slight changes during the first five sessions. Interestingly this response pattern was associated with a low benefit from therapy at post-treatment assessment in all three groups.

In two of the studies mentioned above [4, 8] the authors tried to identify predictors of early response in psychotherapy. Fennel et al. [4] could not find differences between depressive patients responding rapidly to CBT vs. patients who only slightly changed during the first two weeks of treatment in regard to demographics, clinical variables (e.g., duration, severity of symptoms), and antidepressant medication at pre-treatment. Lutz et al. [8] could identify depressive and interpersonal problems, agoraphobic symptoms and somatization, and problems at school or in the job as predictors of the identified patterns of changes in the early phase of therapy.

This summary of studies demonstrates that there is a substantial number of patients who experience an early positive change in therapy before it is completed or therapy techniques are initiated. However this phenomenon of early response has been examined only in a very limited number of groups of patients – mainly in depressive patients [4, 5, 7] or patients with mixed diagnoses [6, 8]. Other clinical samples, in which early response could be of special interest, have not been investigated in regard to this phenomenon. A clinical sample could be patients suffering from syndromes of medically unexplained physical symptoms (MUS).

Medically unexplained symptoms (MUS) are somatic symptoms that cannot or have not been sufficiently explained by organic causes after a thorough physical examination [9]. According to diagnostic criteria of the fourth revision of the Diagnostic and Statistical Manual for Mental Disorders (DSM-IV) [10], the chronic and severely distressing manifestation of MUS is a core characteristic of any one of the following somatoform disorders: somatization disorder, undifferentiated somatoform disorder, and pain disorder. According to the current revision of DSM - DSM-5 [11] – these three diagnostic categories are covered by somatic symptom disorder. Syndromes of MUS are highly prevalent in the general population [12], in primary care [13], and in medical specialist settings [14]. In longitudinal studies a high risk of symptom persistence has been demonstrated [15]. MUS and somatoform disorders are associated with high rates of comorbid mental disorders [16], high levels of symptom-related burden and disability in everyday life functioning [17]. As a consequence of extensive utilization of health care services [18] health care costs are substantially increased in this group of patients. Psychological interventions, especially cognitive behavior therapy, have been demonstrated to be effective in reducing symptom severity but also secondary outcomes (e.g., level of functional disability, depressive and anxiety symptoms) [19, 20].

There are several reasons why early response patterns in somatoform patients could be of special interest. Due to the somatic nature of their complaints patients with somatoform disorders partly attribute their symptoms strongly to biomedical causes [21]. Consequently for a subset of these patients it is difficult to consider psychotherapy as a helpful and appropriate treatment for their complaints. Due to this issue it is not surprising that the physician-patient relationship can be conflict-laden and characterized by problems in communication [22]. A typical feature of such communication problems is that patients appear as demanding or premeditated to pursue certain ideas (especially finding a somatic cause of their symptoms) [23]. In turn physicians can feel pressured or controlled [24]. When patients strongly complain about their symptoms physicians are likely to prescribed somatic interventions [25]. Thus it is not surprising that patients suffering from long-standing MUS often present with a long history of somatic diagnostic procedures and treatments often accompanied by feelings of disappointment and frustration, negative interpersonal relationship experiences, and hopelessness [26]. These negative experiences usually contrast with interpersonal experiences in the early process of psychotherapy and may reduce the rate of early response. To our knowledge there is only one previous study examining the phenomenon of early response in patients with MUS. Lackner, Gudleski, Krasner, Powell, and Katz [27] demonstrated that 30% of patients suffering from a specific functional somatic syndrome characterized by unexplained gastrointestinal symptoms – irritable bowel syndrome (IBS) – fulfilled criteria of rapid response (affirmative response to 2 binary [Yes/No] adequate relief measures of pain and other IBS symptoms; decrease in Irritable Bowel Syndrome Severity Scale [28] total score of ≥50 points after four weeks of treatment; patients who did not meet both of these criteria at week 12 were classified as non rapid responders). Rapid responders had higher symptom severity at baseline but also a better outcome in regard to symptom reduction at post-treatment in comparison to non rapid responders.

Ilardi and Craighead [29, 30] reviewed studies of CBT for depression in regard to common factors and demonstrated that the majority of symptomatic improvement in CBT occurs prior to the formal induction of techniques being specific to CBT. Accordingly the authors assumed that non-specific factors rather than specific techniques explain the efficacy of CBT, in particular by ameliorating patients’ feelings of hopelessness at the beginning of treatment. This explanation underlines the idea that early response patterns in psychotherapy could play a special role in samples of patients with chronic somatoform disorders: New experiences of therapeutic alliance contrast with patients’ previous problematic experiences of interpersonal patient-clinician relationship. This idea can be especially well investigated in the context of the German health-care system which offers patients with mental disorders five therapy-preparing sessions before therapy actually starts. During these sessions actions such as diagnostics, or creating a therapy plan but no specific therapeutic techniques are initiated.

Therefore the *first aim* of the current study was to examine if there are changes in general regarding the severity of somatic symptoms and symptom-related outcomes after 5 therapy-preparing sessions. The *second aim* was to analyze if response scores after these therapy-preparing sessions predict therapy outcomes at the end of therapy, controlling for pre-treatment level of disturbance. The *third aim* was to compare patients reaching a reliable change of symptoms during the early five sessions of therapy with patients who do not reach this reliable change, in regard to their symptom scores at pre-treatment, after the therapy-preparing sessions, and at the end of treatment. Finally the *fourth aim* was to examine if early changes in psychotherapy for somatoform disorders are associated with demographic and clinical variables.

## Methods

### Participants

A total of 106 participants were treated between April 2007 and September 2013 at a psychotherapeutic university outpatient clinic in Germany where a new treatment program for patients with syndromes of MUS was implemented. Patients were recruited via a waiting list for a treatment at the University Outpatient Clinic for Psychotherapy of Johannes Gutenberg-University Mainz (Germany), public media, and flyers which were placed in pharmacies, private practices of general practitioners or different kinds of medical specialists (e.g., orthopedists, gastroenterologists).

Participants being eligible for this study had to fulfill the following inclusion criteria: a) ≥ 3 distressing MUS over the last six months or longer, b) exclusion of medical causes of the symptoms by a physician, c) comorbidity with other mental disorders was allowed, as long as MUS were considered to be the major problem, d) age between 18 and 65 years, and e) sufficient German language skills. Exclusion criteria were a) one or more of the following psychiatric conditions: current severe episode of a major depressive disorder or bipolar disorder, suicidality, eating disorder, substance use disorder, acute psychosis, brain injury, cluster A or B personality disorder, dementia or neurodegenerative disorders, or PTSD, b) use of benzodiazepines or neuroleptics, and c) psychological inpatient treatment during the last 5 years or psychological outpatient treatment addressing MUS during the last 2 years, and d) or submitted retirement request due to MUS.

### Study design and procedure

This study was designed as a randomized controlled trial. Participants were admitted to the study via a two-step procedure (telephone screening, face-to-face diagnostic interview). Eligibility criteria were first checked during the telephone screening. Participants who fulfilled the criteria were asked to complete questionnaires, were invited to a diagnostic interview conducted by trained clinical psychologists based on International Diagnostic Checklists for DSM-IV [31], and were asked to sign informed consent. Excluded participants were informed about other treatment options and were offered registry for regular psychotherapy at the outpatient clinic. Participants who fulfilled inclusion criteria were randomly assigned to either the cognitive behavioral treatment group (CBT) or the waiting list control group (WCL). Patients were assigned to a therapist during the 7 days after randomization had taken place. The treatment started with 5 therapy-preparing sessions followed by 20 sessions of cognitive behavior therapy for somatoform disorders. Participants of the waiting-list control condition (WLC) started the treatment after a waiting period of 10 weeks. Randomization was stratified for sex and symptom-related disability based on the score of the Pain Disability Index (PDI; [32]) at baseline (PDI ≤ 6; > 6). The randomization was conducted by a computer scientist being independent of the trial. Only patients were blinded to the randomization results. Study coordinators and therapists were informed about the randomization results immediately after randomization had been conducted. Diagnostic study personnel were also not blinded regarding random assignments.

Since previous randomized controlled trials (RCTs) comparing CBTs with waiting control groups revealed on average medium between-group effect sizes (f = 0.25, Power [1-β error probability] = 0.80, α = 0.05) [19, 20] we conducted a priori a power analysis with G*Power 3 [33] which resulted in a total sample size of *N* = 90. Under consideration of an average dropout rate of 15% at post-treatment which was found in previous RCTs being comparable to our trial [19, 20], we estimated a total sample size of *N* = 106.

All data were collected via paper-pencil versions of self-rating scales before the face-to-face diagnostic interview (pre-treatment) and follow-up assessments took place after 5 diagnostic, therapy-preparing sessions (FU-5P), at the end of treatment after 20 sessions CBT (FU-20 T), and 1 year after the end of treatment. Unfortunately, there was a substantial problem with implementing the study: An additional assessment at the end of the waiting period in the WCL was missed. Consequently the study design did not allow comparing outcomes of CBT and WCL. For the current analysis we will refer only to the participants of the CBT group. Moreover due to low response rates at the 1-year follow-up we evaluated only data of the pre-treatment assessment, of FU-5P, and FU-20 T. The study was approved by the Ethics Committee of the German Psychological Association (DGPs).

### Cognitive behavior therapy for chronic MUS.

Before CBT started, patients received 5 individual sessions à 50 min which did not encompass therapeutic interventions, but had the following contents: exploration of somatic symptoms and comorbid mental disorders, previous somatic and other treatments, resources and coping strategies, patients’ social background and biography; personality disorder diagnostics with the Structured Clinical Interview for DSM-IV Axis II personality disorders (SCID-II) [34]; behavior analyses; and the generation of a therapy plan. After these 5 preparing sessions the therapy actually started, encompassing 20 individual sessions à 50 min (assigned to 6 modules: therapeutic targets, stress management training, attention refocusing training, cognitive restructuring, changing illness behaviors, explanatory model, and relapse prevention) whose primary aim was not to reduce the severity of the somatic symptom but to improve patients’ quality of life and symptom coping. Table 1 summarizes the treatment protocol which was published elsewhere [35]. All sessions were structured in a similar way: After reflecting on patients’ experiences with therapeutic exercises patients conducted between sessions, the therapeutic topic of the session followed, and finally further therapeutic exercises were planned.

**Table 1:** Contents of 20 sessions of the CBT for patients with somatoform disorders

Module/Session	CBT for chronic MUS
1/1	Therapeutic targets Exploration patient’s illness beliefs; developing therapeutic targets
2/2–3, 16–19	Stress management training Psychoeducation: distress and physical symptoms; stress management and relaxation techniques
3/4–6	Refocusing attention training Psychoeducation: relationship between attention and physical symptoms; focusing exercises to shift attention away from physical symptoms
4/7–11	Cognitive restructuring of symptom-related dysfunctional cognitions Psychoeducation: dysfunctional thoughts and physical symptoms; identifying individuals’ dysfunctional symptom-related thoughts; strategies of reappraisal and cognitive restructuring; behavioral experiments (symptom induction) for questioning dysfunctional symptom-related thoughts
5/12–15	Changing illness behaviors Psychoeducation: illness behaviors and physical symptom; symptom inductions tasks/exposure exercises and cognitive strategies to reduce avoidance behaviors and doctor-shopping
6/20	Explanatory model and relapse prevention Summary of therapy contents in an individual explanatory model of MUS; relapse prevention

*CBT* cognitive behavior therapy, *MUS* medically unexplained symptoms

CBT was conducted by master’s-level clinical psychologists who were either certified CB therapists or in the second year of their postgraduate CBT program. They received specialized training in CBT for somatoform patients and continuous supervision every fourth (therapy-preparing as well as therapy) session. Therapy sessions were recorded and records were discussed during supervision in order to increase therapists’ adherence and competence.

### Measures

#### Primary outcome

In order to assess *somatic symptom severity* as the primary outcome, the Screening for Somatoform Disorders-7 T (SOMS-7 T) [36], a self-rating questionnaire in the form of a list of the 53 physical symptoms of DSM-IV and the 10th revision of the International Classification of Diseases (ICD-10) [37], was administered. The severity over the last seven days of only those symptoms that cannot or not sufficiently be explained medically was rated on a 5-point Likert scale (answering options range between 0 to 4). In the current study the severity index as a mean value of all symptom ratings was used. Internal consistency was demonstrated to be high for the scale (Cronbach’s α = .92) [36], and acceptable in the current sample (α = 0.77).

#### Secondary outcomes

*Depressive symptoms* were assessed with the Beck Depression Inventory-II (BDI-II; 21 items, 4-point answering scale: 0 to 3) [38] which has demonstrated good internal consistency in a previous validation study (α = .86) [38], and also in our sample (α = .83). We used the anxiety subscale (6 items) of the 53-items Brief Symptom Inventory (BSI; answering scale: 5-point answering scale: 0 to 4) [39] in order to assess *anxiety symptoms*. Internal consistency of this subscale was shown to be good (α = .86) [40], but less so in our sample (α = .70). Finally the Illness Attitude Scales (IAS; 29 items, 5-point scale: 0 to 4) [41] were administered in order to assess *illness anxiety* and *illness behavior*. Whereas the illness anxiety subscale demonstrated excellent reliability in a previous validation (α = .85–.87) [42] but also in the current sample (α = .93), the illness behavior subscale had low internal consistency in a previous study (α = .70–.80) [42], and in our sample (α = .66). It seems that the illness behavior subscale usually reveals lower internal consistency values than the illness anxiety subscale which has been shown also in other validation studies (e.g., [43]). This may be explained by the small number of items (6 items in contrast to 17 items of the illness anxiety subscale).

### Definition of early response

Previous studies applied different ways of operationalizing early treatment response. Fennell and Teasdale [4] for example utilized a median split to determine response rate. Renaud et al. [5] defined early response as symptom improvement of at least 50% over a pre-specified number of sessions. A very advanced method was used by Haas et al. [6] who operationalized early response as an average difference between an “expected” recovery rate (which was derived from actuarial data available from a large sample of patients) and the “actual” recovery rate of a patient. Unfortunately such slopes of expected scores are not available for the measures we used. We therefore used the reliable change index (RCI) [44]. Patients who reached the RCI after the five therapy-preparing sessions were defined as early responders. The RCI was calculated by the formula RC = x_2_ – x_1_ /s_diff_, where x_1_ represents a subject’s pretest score and x_2_ its posttest score. S_diff_ is the standard error of the difference between the two test scores, calculated from the standard error of measurement s_E_ by the formula s_diff_ = √ 2 × (s_E_)^2^. For calculating S_E_, the internal consistency (see Cronbach’s α for administered scales under Measures) and the SD of the sample at baseline (see Table 3) were used.

### Data analysis

The statistical analyses were conducted using IBM SPSS Statistics 24. Our analyses are based on intention to treat-analysis for which the multiple imputation procedure offered by IBM SPSS Statistics 20 was used to replace single missing values. The procedure produced five data sets using the monotone multiple imputation algorithm [45]. These five imputed data sets were then analyzed by using standard procedures used for complete data, and then by combining the results across these analyses. *P*-values below an alpha error level = .05 indicated significance.

First we examined if patients who completed questionnaires differed from patients who did not complete questionnaires after the 5 therapy-preparing sessions (FU-5P) and at post-treatment (FU-20 T) in regard to demographic variables (gender, age, education), clinical variables (duration of MUPS, comorbid mental disorder), and self-rating scale pre-treatment scores with Chi^2^-test and *t-*tests.

In order to answer our *first research question* we conducted a repeated measure (rm) analysis of variance (ANOVA) with the innersubject factor “time” (pre-treatment vs. FU-5P). Dependent variables were the SOMS-7 T severity index, mean scores of the BDI-II, BSI anxiety subscale, and IAS illness anxiety and illness behavior subscale. To determine the magnitude of within-group changes, effect sizes using Cohen’s *d* based on the pooled standard deviations (SD) and the corresponding 95% confidence intervals (CI) were reported [46]. According to Cohen’s convention effects of ≥0.10/<0.50 are small, of ≥0.50/<0.80 are medium, and ≥0.80 are large.

In regard to our *second research question* a hierarchal linear multiple regression analysis was conducted separately for each outcome for which a statistically significant change between pre-treatment and the 5th therapy-preparing session had been found (BDI, BSI-anxiety, IAS-illness anxiety, IAS-illness behavior). The predicted variable in each regression analysis was the post-treatment score (FU-20 T) of the outcome respectively. As predicting variables we entered the pre-treatment score of the outcome in the first step of the regression analysis, and additionally the difference score between the pre-treatment and FU-5P score in the analysis’ second step.

Our *third research question* required dividing the sample in two groups, group 1 fulfilled the RCI (RCI+) and group 2 did not fulfill the RCI (RCI-). This group splitting was conducted in regard to each outcome for which a statistically significant change between pre-treatment and the 5th therapy-preparing session had been found (BDI, BSI-anxiety, IAS-illness anxiety, IAS-illness behavior). We then calculated mixed effect 2 (RCI+ vs. RCI-) × 3 (baseline vs. FU-5P vs. FU-20 T) ANOVAs. In order to break up significant main and interaction effects we calculated Bonferroni-corrected post-hoc comparisons of cell means.

Finally, in order to examine the *fourth research question* we conducted bivariate Pearson product-moment or Spearman rank (for sex, educational level, comorbidity) correlation analyses between demographic/clinical variables, pre-treatment scores of self-rating scales and the residual gain score (RGS) based on the difference between pre-treatment and FU-5P. These correlation analyses were conducted only for RGSs based on variables for which a significant change between pre-treatment and FU-5P had been demonstrated (BDI, BSI-anxiety, IAS-illness anxiety, IAS-illness behavior). RGSs were calculated to handle measurement error of repeated administration of the instruments and the initial difference between individuals at pretreatment [47], since pre-analyses showed a high correlation between the pre-treatment score and difference values between baseline and FU-P5 for each outcome (BDI: *r* = .57, *p* < .001; BSI-anxiety: *r* = .79, *p* < .001; IAS-illness anxiety: *r* = .41, *p* = .004; IAS-illness behavior: *r* = .65, *p* < .001). RGSs were calculated by the formula RGS = *z*_2_ − (*z*_1_ * *r*_1,2_), where *z*_2_ is the standardized post-treatment score, *z*_1_ the standardized pre-treatment score, and *r*_1,2_ the Pearson product-moment correlation between raw scores at pre- and post-treatment [47]. RGSs were reversed such that positive scores indicate improvement and negative scores deterioration.

## Results

### Participant characteristics

**Fig. 1:**
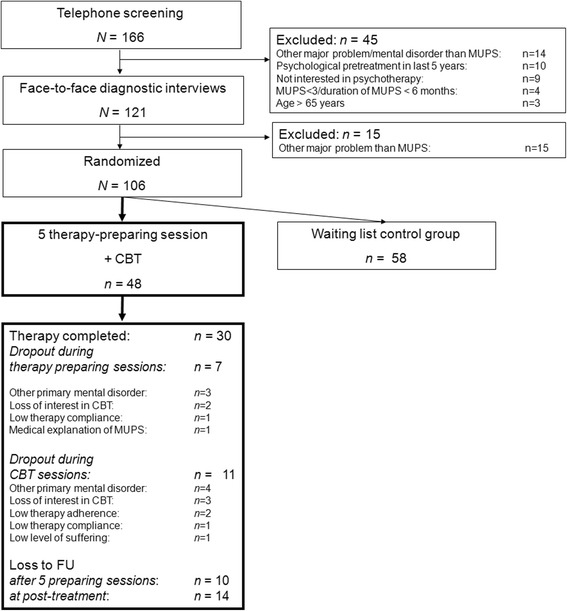
Flow-chart of the RCT

Since only participants from the treatment group were included to the analyses this section will not address participants from the control group. The flowchart in Fig. 1 shows that 48 patients were randomly assigned to the treatment group. The sample was aged on average 40.9 years (*SD* = 14.12, range: 20–65 years) and 64.6% (*n* = 31) were females. Moreover 41.7% had a secondary educational level or lower according to the International Standard Classification of Education (ISCED 97) [48]. In the sample 39 (81.3%) patients fulfilled the criteria of an undifferentiated somatoform disorder, 4 patients (8.3%) of a somatization disorder, and *n* = 5 (10.4%) of a pain disorder. Moreover 50.0% suffered from at least one comorbid disorder. On average individuals in the treatment group suffered 6.59 years (*SD* = 4.70, range: 7 months – 25 years) from their somatic symptoms. Average Body Mass Index (BMI) was 23.11 kg/m^2^ (*SD* = 4.40, range: 16.54–35.66 kg/m^2^). As defined in the exclusion criteria of this trial, patients were not allowed to have psychological inpatient treatment during the last 5 years or psychological outpatient treatment addressing MUS during the last 2 years before starting the current study therapy. The general binary question if participants have ever attended a psychotherapy was answered with “no” by 64.4% (*n* = 31). Pre-treatment scores of self-rating scales are depicted in Table 2.

**Table 2:** Mean values, standard deviations at pre-treament, after 5 therapy-preparing sessions, and after 20 therapy sessions and effect sizes

Outcome	*M* (*SD*)			*d* (95% CI)		
	Baseline	5th session	20th session	FU-1 vs. baseline	FU-2 vs. FU-1	FU-2 vs. baseline
SOMS-7 T	0.62 (0.31)	0.63 (0.38)	0.55 (0.36)	−0.03 [−0.43, 0.37]	0.22 [−0.19, 0.62]	0.21 [−0.19, 0.61]
BDI-II	0.69 (0.35)	0.58 (0.30)	0.48 (0.37)	0.34 [−0.07, 0.74]	0.30 [−0.11, 0.70]	0.58 [0.17, 1.00]
BSI-anxiety	0.95 (0.59)	0.64 (0.43)	0.62 (0.59)	0.60 [0.18, 1.02]	0.04 [−0.36, 0.44]	0.56 [0.14, 0.97]
IAS-illness anxiety	1.65 (0.86)	1.33 (0.81)	1.06 (0.69)	0.38 [−0.02, 0.79]	0.36 [−0.05, 0.77]	0.76 [0.33, 1.18]
IAS-illness behavior	2.40 (0.61)	2.16 (0.53)	1.72 (0.63)	0.42 [0.01, 0.83]	0.76 [0.33, 1.18]	1.10 [0.64, 1.55]

*SOMS-7 T* Screening of Somatoform Disorders-7 T (answering scale: 0 = not at all, 4 = very strong), *BDI-II* Beck Depression Inventory-II (answering scale: 0–3), *BSI* Brief Symptom Inventory (answering scale: 0 = not at all, 4 = very strong), *IAS* Illness Attitude Scales (answering scale: 0 = no, 4 = most of the time)

Of the 48 participants 30 (62.5%) completed the 5 therapy-preparing and 20 therapy sessions, 7 (14.6%) quit therapy during the therapy-preparing sessions, and 11 (22.9%) quit during the 20 therapy sessions. Reasons for therapy dropout are depicted in Fig. 1. Patients who completed therapy did not significantly differ from patients who dropped out of therapy in regard to demographic variables (age, sex, education, BMI), clinical variables (duration of somatic symptoms, comorbid mental disorders, and baseline scores of self-rating scales) (*p* ≥ .165). Moreover, 38 patients who completed questionnaires after the five therapy-preparing sessions did not significantly differ from patients who did not fill in questionnaires (*p* ≥ .085) in regard to the demographic and clinical variables, except for the BMI, *t*(44) = −2.05, *p* = .046. Patients who did not complete the questionnaires had a higher BMI (*M* = 25.55, *SD* = 5.71) than patients who completed the self-rating scales (*M* = 22.43, *SD* = 3.78). A similar result was found when 34 patients who completed questionnaires at post-treatment were compared with 14 patients who did not complete self-rating scales (*p* ≥ .256). There was only a difference in regard to educational level: group of patients who completed self-rating scales at FU-20 T were significantly higher educated than patients who did not complete questionnaires at FU-20 T, Chi^2^(1, *N* = 47) = 5.43, *p* = .025.

### Changes during 5 therapy-preparing sessions

RmANOVAs revealed a significant change between pre-treatment and FU-5P for the BDI, *F*(1,47) = 6.68, *p* = .014, the anxiety subscale of BSI, *F*(1,47) = 9.86, *p* = .004, for the illness anxiety subscale, *F*(1,47) = 13.59, *p* = .001, and illness behavior subscale of IAS, *F*(1,47) = 8.30, *p* = .007, but not for SOMS-7 T, *F*(1,47) = 0.05, *p* = .828. Results indicated that depressive and anxiety symptoms, illness anxiety, as well as illness behavior significantly declined during the first 5 therapy-preparing sessions with small to medium effect sizes (see Table 2), whereas somatic symptom severity did not change. Table 2 summarizes mean values and standard deviations for pre-treatment scores and FU-5P as well as effect sizes.

### Prediction of outcome at the end of therapy by pre-treatment values and changes during therapy-preparing sessions

Hierarchical linear multiple regression analyses were conducted for the outcomes for which we found significant changes during the 5 therapy-preparing sessions: depression, anxiety, illness anxiety, and illness behavior. In all regression analyses the pre-treatment score was a significant predictor of the outcome at the termination of therapy, in the first and the second model of the analysis. The results indicated that high pre-treatment values were associated with high values at the end of treatment. For depression and illness anxiety the differential score between pre-treatment and FU-5P became an additional significant predictor in model 2 and explained additionally 12.1%, F(1,45) = 8.01, *p* = .007, and 10.0%, F(1,45) = 7.46, *p* = .009, respectively of the variance of the outcome at the end of therapy. Results indicated that a reduction of outcome between pre-treatment and FU-5P was associated with lower values on the self-rating scales at FU-20 T. In contrast, for anxiety and illness behavior the differential score between pre-treatment and FU-5P did not become an additional significant predictor in model 2. The differential score explained additionally only 5.0%, *F*(1,45) = 2.97, *p* = .092, and 5.3%, *F*(1,45) = 3.53, *p* = .067, respectively of the variance of outcome at the end of therapy. Test statistics of the regression analyses are summarized in Table 3.

**Table 3:** Test statistics of regression analyses of predictions of outcome at therapy termination by pre-treatment scores and changes during therapy-preparing sessions

	*B*	β	*R* ^*2*^	*F*	*p*
Depression					
Model 1^a^			.183	10.39**	.002
BDI-II (pre-treatment)	.37	.43**			.002
Model 2^b^			.305	10.41**	.001
BDI-II (pre-treatment)	.57	.66***			<.001
BDI-II (∆ FU-5P – pre-treatment)	−.50	−.42**			.013
Anxiety					
Model 1^a^			.161	8.87**	.002
BSI-anxiety (pre-treatment)	.34	.40**			.004
Model 2^b^			.211	5.843**	.006
BSI-anxiety (pre-treatment)	.56	.67*			.018
BSI-anxiety (∆ FU-5P – pre-treatment)	−.26	−.33			.235
Illness anxiety					
Model 1^a^			.291	18.99**	.002
IAS-illness anxiety (pre-treatment)	.44	.54***			<.001
Model 2^b^			.391	16.84***	<.001
IAS-illness anxiety (pre-treatment)	.55	.68***			<.001
IAS-illness anxiety (∆ FU-5P – pre-treatment)	−.40	−.34*			.016
Illness behavior					
Model 1^a^			.271	17.34**	.002
IAS-illness behavior (pre-treatment)	.51	.52***			<.001
Model 2^b^			.323	10.71***	<.001
IAS-illness behavior (pre-treatment)	.70	.71***			<.001
IAS-illness behavior (∆ FU-5P – pre-treatment)	−.32	−.29			.135

*SOMS-7 T* Screening of Somatoform Disorders-7 T, *BDI* Beck Depression Inventory-II, *BSI* Brief Symptom Inventory, *IAS* Illness Attitude Scales

^a^ *df*_n_ = 1, *df*_d_ = 46; ^b^ *df*_n_ = 2, *df*_d_ = 45

* *p* < .05, ** *p* < .01, *** *p* < .001

### Differences RCI+ and RCI-

For the BDI only 2 (4.2%) and for illness behavior subscale of IAS only 1 patient (2.1%) reached a RCI. Consequently, differences between RCI+ and RCI- were analyzed only for the BSI anxiety subscale and the IAS illness anxiety subscale since they were the only outcomes for which a sufficient number of participants reached a reliable change.

**Fig. 2:**
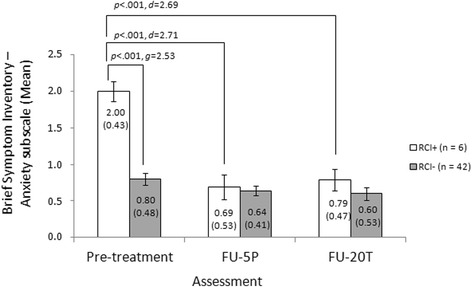
Mean values and standard deviations at pre-treatment, follow-up after 5 therapy-preparing sessions (FU-5P) and 20 therapy sessions (FU-20 T), between-group and inner-subject effect sizes and test statistics for the anxiety subscale of the Brief Symptom Inventory (separately for patients who reached a clinically significant decline of anxiety symptoms after 5 therapy-preparing sessions [RCI+] versus patients who did not [RCI-])

Regarding the BSI anxiety subscale an improvement of at least 0.94 was necessary to reach a reliable change. We observed this reliable change during the 5 therapy-preparing sessions in 6 (12.5%) patients. A mixed-effect 2 × 3 ANOVA resulted in a significant main effects of time, *F*(2,92) = 23.49, *p* < .001, and group, *F*(1,46) = 10.21, *p* = .003, and a significant time*group interaction effect, *F*(2,92) = 13.38, *p* < .001. Bonferroni-corrected post-hoc comparisons were calculated in order to break up the significant interaction effect. They showed that RCI+ group had significantly higher anxiety values than the RCI- group at pre-treatment, *p* < .001, *g* = 2.53 (95% CI: 1.53, 3.52), whereas at FU-5P and FU-T20 no significant between-group differences were found (*p* ≥ .349). In the RCI+ group a significant decline of anxiety symptoms between pre-treatment and FU-5P, *p* < .001, *d* = 2.71 (95% CI: 0.81, 4.62) or FU-20 T, *p* < .001, *d* = 2.69 (95% CI: 0.79, 4.58) was found. The remaining inner-subject cell comparisons did not reveal further significant effects (*p* ≥ .069). Mean values, standard deviations, and effect sizes are summarized in Fig. 2.

**Fig. 3:**
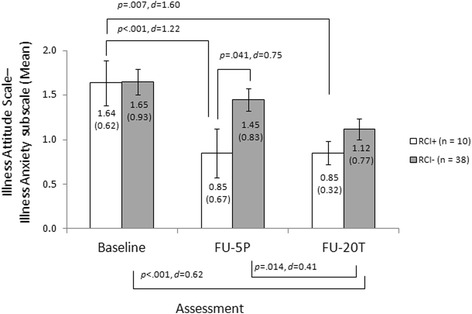
Mean values and standard deviations at pre-treatment, follow-up after 5 therapy-preparing sessions (FU-5P) and 20 therapy sessions (FU-20 T), between-group and inner-subject effect sizes and test statistics for the illness anxiety subscale of the Illness Attitude Scales (separately for patients who reached a clinically significant decline of illness anxiety symptoms after 5 therapy-preparing sessions [RCI+] versus patients who did not [RCI-])

Regarding the IAS illness anxiety subscale an improvement of 0.64 points indicated a reliable change. During the 5 therapy-preparing sessions 10 patients (20.8%) reached the RCI. A mixed-effect 2 × 3 ANOVA resulted in a significant main effect of time, *F*(2,92) = 16.31, *p* < .001. However the main effect of group, *F*(1,46) = 1.40, *p* = .244, did not reach significance, and also the time*group interaction effect slightly failed to reach significance, *F*(2,92) = 3.01, *p* < .054. Bonferroni-corrected post-hoc comparisons showed only one significant between-group difference, at FU-5P: RCI+ had significantly lower illness anxiety scores than RCI- group, *p* = .041, *g* = 0.75 (95% CI: 0.04, 1.46). Moreover post-hoc comparisons showed that in the RCI+ group a significant decline of illness anxiety took place between pre-treatment and FU-5P, *p* < .001, *d* = 1.22 (95% CI: 0.20, 2.25), and between pre-treatment and FU-T20, *p* = .007, *d* = 1.60 (95% CI: 0.48, 2.72). In contrast in the RCI- group the significant decrease of illness anxiety was found between FU-5P and FU-20 T, *p* = .014, *d* = 0.41 (95% CI: -0.05, 0.87) and between pre-treatment and FU-20 T, *p* < .001, *d* = 0.62 (95% CI: 0.15, 1.09). Mean values, standard deviations and effect sizes are summarized in Fig. 3.

### Relationships between changes during the 5 therapy-preparing sessions and demographic or clinical variables at baseline

We calculated RGSs based on the difference between pre-treatment and FU-5P for variables which demonstrated a significant change the five therapy-preparing sessions: BDI, BSI anxiety subscale, IAS illness anxiety and illness behavior subscale. The Pearson product-moment and Spearman rank correlation coefficients are summarized in Table 4. None of the correlation coefficients reached significance.

**Table 4:** Pearson product-moment and Spearman rank correlation coefficients between RGS scores and demographic and clinical variables, and baseline scores of self-rating scales

	*r* (*p*)			
	RGS BDI	RGS BSI-A	RGS IAS-IA	RGS IAS- IB
Sex^a^	.01 (.957)	−.05 (.763)	−.11 (.453)	.01 (.946)
Age	.18 (.240)	−.06 (.717)	.03 (.825)	.08 (.608)
Education^a^	−.11 (.495)	.02 (.989)	.04 (.777)	.06 (.719)
BMI	−.03 (.832)	.03 (.861)	.17 (.282)	−.20 (.309)
Duration of somatic symptoms	−.22 (.178)	−.23 (.162)	−.23 (.129)	−.09 (.563)
Comorbidity^a^	−.16 (.279)	−.16 (.297)	.23 (.129)	.10 (.541)
SOMS-7 T (Baseline)	−.12 (.441)	−.04 (.792)	.09 (.537)	−.14 (.383)
BDI (Baseline)	---	−.01 (.950)	.05 (.767)	−.01 (.943)
BSI-A (Baseline)	.11 (.473)	---	.09 (.545)	−.00 (.989)
IAS-IA (Baseline)	.14 (.383)	−.06 (.685)	---	−.03 (.859)
IAS-IB (Baseline)	−.03 (.862)	.02 (.895)	.08 (.597)	---

*SOMS-7 T* Screening of Somatoform Disorders-7 T, *BDI* Beck Depression Inventory-II, *BSI-A* Brief Symptom Inventory – Anxiety subscale, *IAS-IA/−IB* Illness Attitude Scales – illness anxiety subscale/illness behavior subscale, *BMI* Body Mass Index

^a^ Spearman rank correlation coefficients

## Discussion

The central aims of the current study were first to examine early symptom changes in patients with medically unexplained symptoms (MUS) during five therapy-preparing sessions. Secondly, we wanted to find out if these early changes are predictors of therapy outcome at therapy termination, after controlling for pre-treatment scores. The third aim was to investigate differences between patients who reached a reliable change after the therapy-preparing phase with patients who did not. Finally we examined if early changes in CBT for somatoform disorders are associated with demographic and clinical variables. Most important findings are summarized and discussed in the following.

The first important result is that although the severity of patients’ somatic symptoms did not change during the therapy-preparing phase, patients experienced a significant decline of depressive symptoms, anxiety, illness anxiety and illness behavior. In comparison to other studies of early response in psychotherapy the rates of responders was however rather small. The highest rate of patients who reached a reliable change in the first 5 sessions was found for illness anxiety (10 of 48 patients, 20.8%) and for anxiety (6 of 48 patients, 12.5%). Inner-subject effect sizes of these early changes were small to moderate across outcomes. Our findings are similar to a previous study which examined early response in patients suffering from irritable bowel syndrome (IBS) [27]. Authors of this previous study found a similar rate of early response (30% of the participants fulfilled criteria of early response after the 4th therapy week) [27]. In contrast to our study, Lackner, Gudleski, Krasner, Powell, and Katz [27] found however an early response effect also in regard to somatic symptoms. However it has to be considered that in the mentioned study [27] patients received the therapeutic interventions immediately the first therapy session. There were no therapy-preparing sessions like in our study.

In studies based on patients with other mental disorders early response rates are higher. For example in the study by Fennell and Teasdale [4] 47.1% of a depressive sample showed a decrease of depressive symptoms of 50% or more. In the study by Renaud et al. [5] 31.0% had an improvement of at least 50% during the first two therapy sessions. However it has to be considered that meta-analyses demonstrated CBT to be not as effective for MUS [19] as for depression [49]. Moreover our results are difficult to compare with other previous studies since they do not only differ in regard to participants’ diagnoses but also in regard to the number of sessions being considered for the definition of early response (e.g. 2 sessions in the studies by Fennell and Teasdale [4] and Renaud [5] vs. 5 sessions in our study) and the definition of early response (e.g. reaching RCI in our sample vs. a symptoms’ decrease of 50% or more in the study by Fennell and Teasdale [4]). Erekson, Lambert, and Horner [50] have shown that the different methods of defining dramatic change produce large discrepancies in rates of individuals meeting criteria. These ranged from 42% to 5% based on a single measure and a single sample of outpatients with mixed diagnoses. Furthermore, our study differed from previous studies in regard to contents of the early sessions. Whereas in our study the first five sessions had a therapy-preparing character and did not include any CBT interventions, in other studies therapeutic interventions already took place during the early treatment phase.

An important question is how this change in affective-cognitive and behavioral outcomes in a sample of patients with long-standing and distressing MUS can be explained. It could be speculated if this result indicates a kind of “placebo effect”. The concept of “placebo effect” comes from pharmacological research and is by definition “the effect that occurs following administration of a placebo, that is, an inert treatment” [51]. However this definition is very restrictive when placebo-like effects in which no placebo is given are considered [52] and can be applied to psychotherapy research only to a limited extend. These effects are rather attributable to the impact of the context factors surrounding the treatment [51] such as different kinds of patients’ expectations [53]. Accordingly, Lambert [54] assumes that early response patterns in psychotherapy are rather evidence for common factors than a “placebo effect” in its original medical meaning. Common factors are those dimensions of the treatment setting (e.g., therapist, client) which are not specific to a particular therapeutic technique. As already mentioned in pharmacotherapy early response is rather considered as a “premature” change in symptomatology associated with a final negative therapy outcome and risk of relapse [2, 3]. In contrast, in psychotherapy previous research however has already demonstrated that across different forms of therapy early response seems to be associated with a positive therapy outcome at termination and follow-up [6, 7].

Ilardi and Craighead [29] examined several studies of CBT for depression in regard to early responses. They explained the phenomenon by the impact of common factors such as the formation of a collaborative therapeutic alliance, the scheduling of frequent and regular appointments at a designated treatment setting, the formal presentation of a treatment rationale, and the introduction of an explicit procedure touted as integral to the recovery process – this means common factors which cannot be attributed to specific therapy techniques. These assumptions would fit very well with our findings. After many years of repeated, mainly ineffective medical diagnostic procedures and treatments associated with the experience of disappointment and frustration as well as the repeated experience of less validating communication with the clinician, it can be assumed that the first psychotherapy sessions are a completely new experience to the prototypic somatoform patient. In CBT for somatoform patients these first therapy-preparing sessions are usually filled with actively listening to the patient and validating the suffering associated with somatic complaints [55] in order to establish a trustful patient-therapist relationship. The patient shall feel understood and taken seriously. Moreover patients experience a kind of reassuring effect during these first sessions which could be based on simply knowing that they will see a professional that is there to help them every week. This could contribute to reducing their anxiety. However at it has been demonstrated in previous research this effect of reassurance would likely not last in the absence of an actual therapy – especially in regard to illness anxiety. This could be explained by the result of another study which demonstrated that patients with MUS (in contrast to patients with depression and healthy subjects) remembered a higher likelihood for medical explanations for their symptoms and reported more concerns about their health state [56].

The aim of these first therapy-preparing sessions is to provide the patient with the expectation that psychotherapy could be a helpful intervention. Moreover patients’ biomedical causal symptom attributions are slowly extended to psychosocial factors. It would make sense that this new interpersonal experience can have a strong impact especially on cognitive-affective variables such as anxiety and depressive symptoms. Although we cannot make any statements about which processes exactly cause changes in these first five sessions, this would be an important question for future research. In CBT for depression Fennell and Teasdale [4] have already found out that early responders engage in therapy in a different manner than later responders. For example early responders appeared to proceed from one problem to the next in therapy in a sequential pattern, whereas delayed responders continued to revisit the same therapy topic across sessions.

A second important finding is that early improvements of depressive symptoms and illness anxiety during the 5 therapy-preparing sessions are predictors of a better outcome at the end of therapy, after controlling for pre-treatment scores. This result is in accordance with the predictive value of early response patterns demonstrated for IBS [27] and other mental disorders [4, 6]. Haas et al. [6] for example found the same pattern of results in a large sample of psychotherapy outpatients with mixed diagnoses. Fennell and Teasdale [4] demonstrated similar predictions of the outcome of the CBT by early response in a sample of depressive patients.

The third important finding of our study refers to the maintenance of symptom improvement after 5 therapy-preparing sessions until the termination of therapy. In regard to anxiety and illness anxiety we identified a sufficient number of patients who reached a reliable change after the therapy-preparing phase. Contrasting them with patients who did not reach the RCI resulted in two important findings. First, between-group differences were dependent on the kind of outcome. Regarding anxiety, especially patients with high pre-treatment scores showed a strong decrease of anxiety symptoms and maintained this until the termination of therapy. They differed from patients who did not reach the RCI, in regard to pre-treatment scores but not in regard to scores after the therapy-preparing sessions and at the end of therapy. In the contrary for illness anxiety there was another pattern. Patients who reached RCI during the therapy-preparing sessions maintained this improvement until the end of therapy. Patients who did not reach RCI during the 5 preparing sessions improved significantly during the 20 therapy sessions. Both groups only differed significantly after the five therapy-preparing sessions, but not at pre-treatment or at therapy termination. Secondly, for both outcomes early response could be maintained until the end of therapy. This has also been demonstrated for IBS [27] and depressive [4, 5] as well as samples of patients with mixed diagnoses of mental disorders [6]. In contrast to the mentioned studies we could however not demonstrate in our somatoform sample that patients with an early response have a better outcome at therapy termination. However our results are just preliminary and are based on very low sample sizes. Therefore they have to be interpreted cautiously.

A final important finding of our study is that we could not identify any demographic or clinical variables being associated with early changes in therapy. These zero-order correlations have also been demonstrated in a sample of depressive patients receiving CBT in a previous trial [4]. However we could show that higher pre-treatment values on a self-rating scale were significantly associated with bigger changes on this scale after the five therapy-preparing sessions. This finding is similar to results of a study examining early response in IBS [27]. In our study the highest correlation was found in regard to anxiety: high pretreatment anxiety scores were associated with a stronger decline of anxiety in the therapy-preparing phase. This result can be possibly explained since these five early sessions are filled with establishing a therapy alliance. The bonding experience can probably have an anxiolytic effect on patients, for example by providing them with reassurance or emotional support and empathy [57]. Of course, it can be assumed that early improvement of symptoms can also have a reverse effect and can improve therapy alliance [58]. Besides the study by Fennell and Teasdale [4] mentioned above, there is only one further study where correlates of early response were investigated. Lutz et al. [8] identified depressive and interpersonal problems, agoraphobic and somatization symptoms, and problems at school or in the job as predictors.

To our knowledge the current study is the first trial which provides important findings in regard to the role of early response in the psychotherapy of somatoform disorders characterized by multiple MUS. For the interpretation of our findings the following limitations of our study however should be considered. First of all, sample size was small and missing rates were high. Although we analyzed differences regarding baseline variables between patients who dropped out and those who completed therapy, we failed to assess other important variables (e.g. pre-treatment therapy motivation) which could be associated with post-treatment results and which could account for dropout rates skewing our findings. Another limitation was that we assessed therapy progress only three times (pre-treatment, FU-5P, and FU-20 T). Process measures which are filled in by patients would be more appropriate in order to examine early response patterns. Moreover due to the failures in the implementation of the study we were not able to consider the control group in our analyses and to compare the control with the CBT data. However we can at least conclude from previous intervention studies that the kind of CBT we provided to our patients has been proven to be effective in comparison to a wait-list control groups [19, 20].

## Conclusions

It is important to emphasize that finally it should not be concluded from our data that “just listening” and “validating symptom-related distress” is sufficient to treat patients with chronic MUS. It is not realistic to expect that the kinds of problems of patients who suffer over many years from distressing and impairing somatic symptoms can be solved within five sessions and that these effects are long-lasting. Our data should be rather interpreted as evidence that changes during these early sessions have a strong predictive value for long-term outcomes in somatoform patients. Although we do not know exactly which mechanisms cause early response patterns it makes sense to assume that new experiences in the therapeutic relationship can already have healing effects in patients with MUS, especially under consideration of patients’ typical previous interpersonal experiences in patient-clinician relationships. This assumption would especially fit specific early responses in regard to affective-emotional variables in contrast to somatic symptom severity. Wampold [59] postulates in his contextual model of psychotherapy that an initial bond between patient and therapist is an important basis for making therapy work and for initiating important therapeutic processes – such as the stepwise extension of MUS-patients’ biomedical causal symptom attributions to psychosocial factors.

Future studies are needed to replicate our findings with larger sample sizes and including active control conditions. These studies should also apply assessments not only after single but after every session in order to monitor the early response pattern and therapy process session by session. Moreover an important conclusion is that future research should not only focus on somatic symptom severity, as it has happened in almost all previous intervention studies on somatoform patients [19, 20], but also include symptom-related outcomes. It has to be emphasized that reducing symptom severity is in fact not the primary aim of CBT for somatoform disorders but rather improving symptom coping. In general it seems that research on early response patterns in psychotherapy should not only focus on primary outcomes [e.g., 4, 5, 7], since these patterns can possibly vary dependent on which type of outcome is considered. We implemented all mentioned issues which are important for future research on therapy processes in CBT for MUS in a further large multicenter RCT in which we compare a conventional CBT with a CBT enriched with emotion regulation strategies for somatoform patients. Further details of this study can be taken from the study protocol [60]. We hope that we can move forward with gaining more insight into early response patterns and its mechanisms in somatoform patients.

## Abbreviations

(rm)ANOVA: (repeated measure) analysis of variance
BDI-II: Beck Depression Inventory-II
BMI: Body Mass Index
BSI: Brief Symptom Inventory
CBT: Cognitive-behavior therapy
DSM-IV/−5: Diagnostic and Statistical Manual for Mental Disorders, 4th/5th revision
FU-5P/−20 T: Follow-up assessment after 5 therapy-preparing sessions/20 therapy sessions
IBS: Irritable bowel syndrome
ICD-10: International Classification of Diseases, 10th revision
MDD: Major depressive disorder
MUS: Medically unexplained symptoms
RCI: Reliable change index
RCI+/−: Subgroup which fulfilled/ did not fulfill the reliable change index
RCT: Randomized controlled trial
RGS: Residual gain score
SOMS-7 T: Screening for Somatoform Disorders-7 T
